# Rectus Abdominis Musculocutaneous Flap With Supercharging for Reconstruction of Extensive Thoracic Defect Due to Deep Sternal Wound Infection: A Case Report

**DOI:** 10.7759/cureus.25862

**Published:** 2022-06-12

**Authors:** Keisuke Shimbo, Haruka Kawamoto, Isao Koshima

**Affiliations:** 1 Plastic and Reconstructive Surgery, Hiroshima Prefectural Hospital, Hiroshima, JPN; 2 Plastic Surgery, International Center for Lymphedema, Hiroshima University Hospital, Hiroshima, JPN

**Keywords:** transverse cervical artery, thoracic defect, supercharging, rectus abdominis musculocutaneous flap, deep sternal wound infection

## Abstract

Deep sternal wound infection is a serious postoperative complication of cardiac surgery and often requires flap reconstruction. Herein, we report a case of deep sternal wound infection with an extensive thoracic defect that was successfully treated using a modified technique. This technique, defined as “supercharging,” anastomoses the deep inferior epigastric artery and vein of pedicled rectus abdominis musculocutaneous flap to the transverse cervical artery and external jugular vein, respectively. The transverse cervical artery is an easily accessible and reliable recipient vessel. Therefore, we recommend that our technique be used, especially in cases of deep sternal wound infection with extensive thoracic defects.

## Introduction

Deep sternal wound infection (DSWI) is a lethal postoperative complication of cardiac surgery with a reported incidence of 0.5%-6% [1]. Flap reconstruction with the pectoralis major, rectus abdominis, latissimus dorsi, and omental flaps is conventionally performed for DSWI [1-3]. Most DSWI, depending on the defect size and location, are adequately covered with these flaps; however, reconstruction is challenging for DSWI with extensive thoracic defects. Previous studies have recommended omental flaps for DSWI with extensive thoracic defects, although this procedure has some limitations [1-3].

Recently, the techniques of anastomosing the deep inferior epigastric artery and vein of pedicled rectus abdominis musculocutaneous flap (RAMF) to the intercostal artery and vein [4] and using free flaps [5,6] have been reported as alternative procedures. We have improved the technique of adjunctive anastomosis of the deep inferior epigastric vessels of pedicled RMAF for a safe reconstruction of DSWI with an extensive thoracic defect; herein, we report our technique.

## Case presentation

A 74-year-old man with a history of type 2 diabetes and tobacco smoking underwent aortic valve replacement with a prosthetic valve (Trifecta™ Valve, Abbott, Japan) and ascending aorta replacement with a prosthetic graft (J-graft Shield Neo, Japan Lifeline, Japan) for severe aortic valvular stenosis and ascending aortic aneurysm, respectively. One month postoperatively, he developed a swelling with purulent drainage on the chest wall. A computerized tomography scan showed sternal dehiscence and fluid accumulation involving the anterior chest wall. On the following day, the sternal hardware was removed, and thorough wound debridement was performed; these procedures revealed DSWI with complete sternal osteomyelitis. Culture from the sternal abscess confirmed Staphylococcus aureus infection. Antibiotic therapy was initiated with intravenous tazobactam-piperacillin. Negative pressure wound therapy with instillation and dwell time (V.A.C.ULTA™ Therapy unit, KCI, Japan) was administered with the following settings: a pressure setting of -100 mmHg and instillation of normal saline, and a dwell time of 20 min every six hours. The patient’s general condition improved, and the signs of infection gradually decreased.

**Figure 1:**
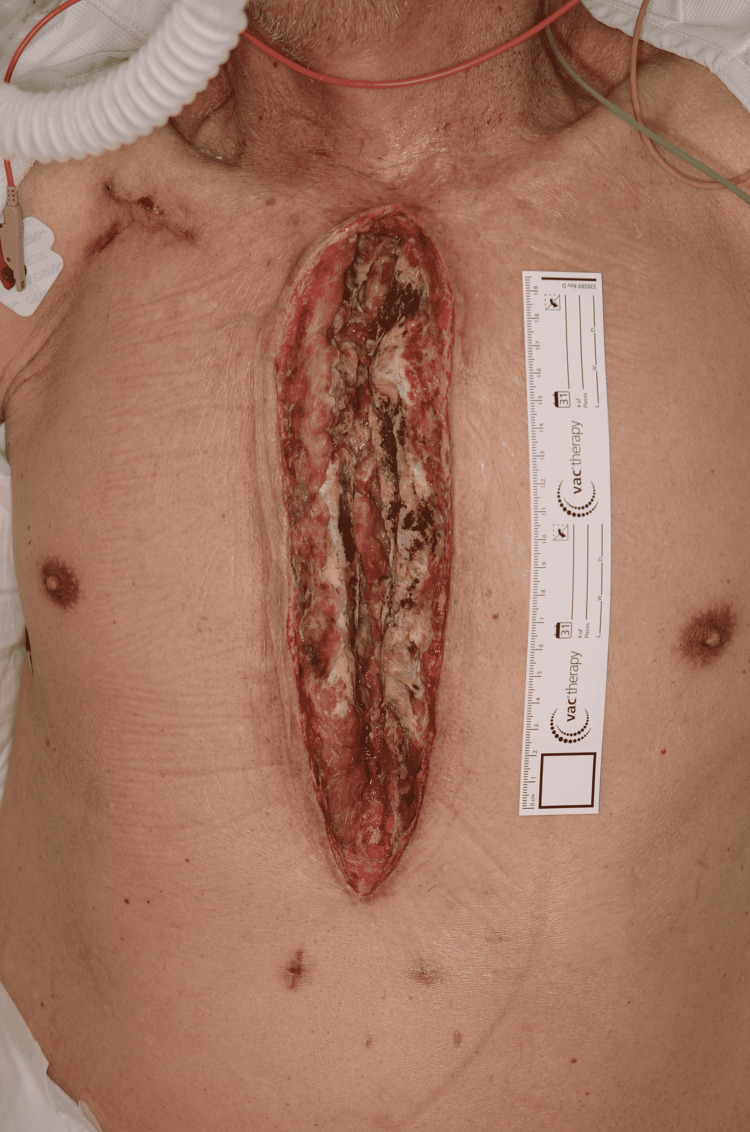
At the time of plastic surgery consultation. DSWI with osteomyelitis of the entire sternum and a 27-cm × 7-cm thoracic defect.

Subsequently, plastic surgeons were consulted for reconstructive surgery (Figure 1).

**Figure 2:**
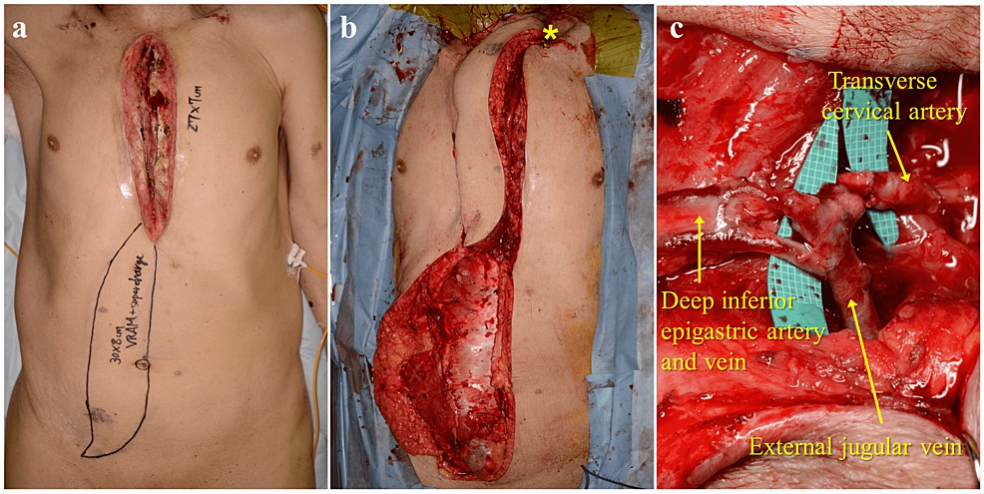
Intraoperative photographs. (a) The rectus abdominis musculocutaneous flap (RAMF) design. (b) The RAMF is rotated 180° and transferred to the thoracic defect. (*) Anastomosis site. (c) Enlarged photograph of the anastomotic site.

Twenty-three days after the first debridement, an additional debridement without sternal fixation was performed (thoracic defect: 27 cm × 7 cm). A pedicled vertical RAMF (30 cm × 8 cm), including the deep inferior epigastric vessels, of a maximum length, was raised following standard procedure [4]. Subsequently, the RAMF was rotated 180° and transferred to the thoracic defect. The deep inferior epigastric artery and vein of the RAMF were anastomosed to the transverse cervical artery and external jugular vein, respectively; this technique was defined as “supercharging” (Figure 2).

**Figure 3:**
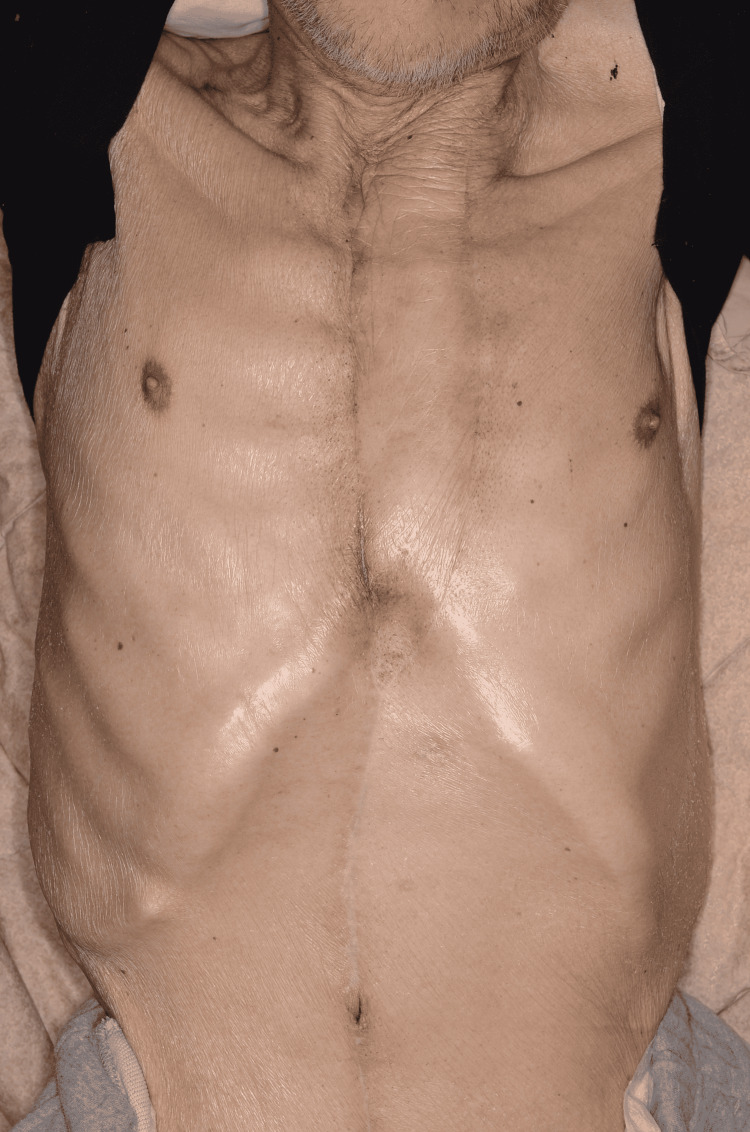
One year after flap reconstruction.

Closed suction drains were placed under the RAMF and at the donor site, and the wound was closed. Although postoperative hematoma was observed, the patient recovered without any other complications. At one year follow-up, there was no recurrence of DSWI (Figure 3).

## Discussion

Pedicled RAMF has been recommended only for reconstruction for caudal defects of DSWI due to inadequate intraflap circulation from the superior epigastric vessels [1]. “Supercharging” effects vascular augmentation to the distal end of the RAMF, allowing coverage of extensive thoracic defects.

The highlight of our “supercharging” technique is anastomosing the deep inferior epigastric vessels of the RAMF to the transverse cervical artery and external jugular vein. Li et al. reported a case series of reconstructive surgery using pedicled RAMF for DSWI; however, only 33% included “supercharging” to the intercostal artery and vein [4]. In studies reporting the use of free flaps, only 20% were anastomosed to the intercostal vessels [5], and most flaps used arteriovenous loop anastomoses to the distant recipient vessels, such as the subclavian vessels [5,6]. Owing to widespread inflammation in DSWI, intercostal vessels may not be available as recipients; thus, they require an arteriovenous loop. DSWI inflammation rarely extends to the transverse cervical artery; thus, this can be safely used as a recipient's vessel to adequately cover thoracic defects without an arteriovenous loop.

However, our technique may not be feasible when both the internal mammary arteries are harvested and unusable. In such cases, a free flap is an option; however, a long vertical flap, as in our case, is unviable due to a single source of blood supply.

## Conclusions

We recommend that our technique be exclusively used for DSWI with extensive thoracic defects since the transverse cervical artery is an accessible and reliable recipient vessel. There is no established consensus yet on a definitive treatment methodology, and further study with an adequate sample size is needed for validation; however, our modified technique may be a viable alternative to those previously reported.
